# *Caulerpa racemosa* extract inhibits HeLa cancer cells migration by altering expression of epithelial-mesenchymal transition proteins

**DOI:** 10.3389/fchem.2022.1052238

**Published:** 2022-11-21

**Authors:** Happy Kurnia Permatasari, Ektina Naura Barbara Ulfa, Vanessa Pradna Adyana Daud, Hikmawan Wahyu Sulistomo, Fahrul Nurkolis

**Affiliations:** ^1^ Biochemistry and Biomolecular, Faculty of Medicine, Brawijaya University, Malang, Indonesia; ^2^ Biomedical Science Study Program, Faculty of Medicine, Brawijaya University, Malang, Indonesia; ^3^ Medical Programme, Faculty of Medicine, Brawijaya University, Malang, Indonesia; ^4^ Pharmacology, Faculty of Medicine, Brawijaya University, Malang, Indonesia; ^5^ Biological Sciences, State Islamic University of Sunan Kalijaga (UIN Sunan Kalijaga), Yogyakarta, Yogyakarta, Indonesia

**Keywords:** anticancer, HeLa cell, *Caulerpa racemosa*, anti-metastatic, epithelial-mesenchymal transition, Snail, Vimentin, E-cadherin

## Abstract

**Introduction:** Cervical cancer is caused by persistent infections of human papillomavirus types 16 and 18. Also, it is classified as a malignancy since it is able to spread itself to other sites and form a metastasis. Lymph nodes metastasis is an important factor related to cervical cancer survival. The previous study reported that *Caulerpa racemosa* has an anti-cancer effect by inducing apoptosis by inhibiting p53 protein degradation in HeLa cancer cells. In this study, we conducted a follow-up test to determine the anticancer effect of *Caulerpa racemosa* as an antimetastatic agent on HeLa cancer cells.

**Methods:** A true experimental study with a post-test-controlled group design was carried out on four groups of HeLa cell cultures by presenting different concentrations of *Caulerpa racemosa* extract. Moreover, to identify the antimetastatic effect, HeLa cells treated with *Caulerpa racemosa* extract were subjected to the woud healing scratch test and immunofluorescence staining assays. Data analysis was gained with qualitative and quantitative approaches. Quantitative methods such as One-way analysis of variance, Tukey’s multiple comparison test, and Pearson’s correlation were conducted.

**Result:** We found that *Caulerpa racemosa* significantly inhibit HeLa cells wound healing migration. We also demonstrated the effect of *Caulerpa racemosa* in downregulating Snail and Vimentin protein expression and upregulating E-Cadherin protein expression.

**Conclusion:** *Caulerpa racemosa* extract inhibits HeLa cancer cells migration by altering important regulator proteins expressions of epithelial-mesenchymal transition pathways.

## Introduction

Cervical cancer is a malignant disease of squamous cells in the cervix (Sulistiowati and Sirait, 2014). This cancer is the second most common type of cancer suffered by women in the world and Indonesia (Sulistiowati and Sirait, 2014; Sung et al., 2021). In 2020, the new cases of cervical cancer in Indonesia were 36,633 cases (17.2%) with a prevalence of 92,930, and the casualty of cervical cancer in Indonesia is the third highest number, after lung and breast cancer, which is 21,003 (9%) (Globocan, 2020). The main cause of cervical cancer is persistent infection with the Human Papilloma Virus (HPV), especially types 16 and 18 (Angsar and Prawiroharjo, 2016). The viral infection causes the rate of proliferation and apoptosis of cells to become unbalanced. Oncogenic signaling from the HPV virus, especially E6 and E7, affects the function of the p53 and pRb genes that act as tumor suppressor genes which affect endothelial growth factor (EGF) signaling (Prihastuti, 2010).

The main reason for the high prevalence and mortality rate in cervical cancer is the metastatic of cancer cells to other parts of the body. The occurrence of cancer cell metastatic cannot be separated from the Epithelial to Mesenchymal Transition (EMT) process, especially type 3 EMT (Qureshi et al., 2015). EMT is a process that causes loss of adhesion and polarity of epithelial cells initially attached to the basement membrane to become cells with a mesenchymal phenotype (Micalizzi et al., 2010; Jaganathan and Supriyanto, 2012). Changes in the properties of these epithelial cells cause the cells to become more motile and invasive (Qureshi et al., 2015). One of the factors that induce the occurrence of EMT is Snail-1 (Snail). Snail is a transcription factor that has a SNAG domain and four functional zinc-fingers. Snail induces EMT through several mechanisms, such as suppressing the expression of E-Cadherin, which plays a role in cell adhesion and upregulation of mesenchymal phenotypic markers such as Vimentin (Wang et al., 2013; Kaufhold and Bonavida, 2014). Thus, the induction of EMT plays an important role in increasing cancer cell’s development and metastatic ability (Micalizzi et al., 2010).

There have been several cancer treatments options in recent years, such as surgery, radiation, and chemotherapy. Only one-third of cancer patients are estimated to be cured with surgery or radiation therapy, and in cancers that have spread, need chemotherapy as a systemic treatment option (Arifianti et al., 2014). These cancer treatment options can also cause major side effects and are relatively expensive. Thus, it is necessary to develop and find new medicines derived from natural ingredients which are abundant and relatively affordable (Wangchuk, 2018).

Seaweed is one of the marine organisms that contains many essential bioactive nutrients such as antioxidants, anticancer, anti-inflammatory, anticoagulant, and antidiabetic (Sanjeewa et al., 2018; Kumar et al., 2021). Sea grapes (*Caulerpa racemosa*) are Chlorophyta seaweed or green algae which exist in Indonesian water, and their utilization has not been optimaly. *C. racemosa* contains essential secondary metabolites, such as caulerpin, caulerpenne (Cyn), caulersin and racemosin C, saponins, flavonoids, tannins, phenols, sulfated polysaccharides, (Meyer and Paul, 1992; Tapotubun et al., 2016; Kumar et al., 2018; Tanna et al., 2018). Caulerpenyne (Cyn) can modify microtubule tissue and has bioactivity against human cell lines, so has a big potential as anticancer, antitumor, and antiproliferative (Chew et al., 2008). In addition, Cyn in *C. racemosa* can inhibit oxidative phosphorylation and interfere with the function of mitochondria which ends in cell death (Yu et al., 2017; Tanna et al., 2020). Recent research also evaluates the potential of *C. racemoca* as an anticancer in HeLa cells. Also, it showed that *C. racemosa* extract with various concentrations significantly increased the expression of pro-apoptotic protein BAX, cleaved caspase-3, total apoptosis, and decreased HeLa cells viability (Permatasari et al., 2022a). This potential is a concern for researchers to examine the health benefits of sea grapes further, especially as cancer drug candidates.

The anticancer potential of *C. racemosa* samples collected from different regions has been investigated in certain cell lines, and the results show the anticancer activity of *C. racemosa* in several cancer markers. However, there are still few studies that discuss the effect of *C. racemosa* on metastatic cancer activity. Therefore, considering the urgency of cervical cancer metastases, we are interested in exploring and developing the potential of natural *C. racemosa* as an anticancer with a therapeutic target of inhibiting cell metastasis in HeLa cervical cancer cells. The results of this study are expected to gain new findings about the potential of natural ingredients as alternative candidates for effective cancer therapy by altering migration and expression of Snail, Vimentin, and E-Cadherin in HeLa cancer cells.

## Materials and methods

### HeLa cell culture

HeLa cervical cancer cells were obtained from the Biomedical Laboratory, Faculty of Medicine, Universitas Brawijaya, Malang, East Java, Indonesia. Cells were cultured in Dulbecco’s modified Eagle’s medium (DMEM) (Invitrogen, Massachusetts), fetal bovine serum (FBS 10%), antibiotics (100 L/ml-penicillin, 100 L/ml Streptomycin), pH 7.2–7.4. HeLa cells were harvested routinely with trypsin—EDTA solution (Permatasari et al., 2021).

### *Caulerpa racemosa* extract preparation

Being Obtained from the shallow waters of Mantehage, North Sulawesi, Indonesia (1°45′47″N 124°43′51″E). Botanical identification and authentication were confirmed at the Pharmacology Department, Faculty of Mathematics and Natural Sciences, Sam Ratulangi University, Indonesia. The finalization of the extract was conducted at the Biochemistry Laboratory, Faculty of Medicine, Universitas Brawijaya, Malang. The coarse powder (1,000 g) was macerated in 96% ethanol and evaporated in an oven at 40°C to produce a thick extract (which resulted in 34% yield) and dissolve the extract using DMSO. In our previous study (Permatasari et al., 2022b), the metabolite profile content of *Caulerpa racemosa* extract has been observed using liquid chromatography–mass spectrometry (LC-MS) and obtained a mass of 398,13,278 (397,12,257 m/z) which indicates the presence of the antioxidant compound caulerpin in *Caulerpa racemosa* extract. This further research proves that the content of secondary metabolites in *Caulerpa racemosa* extract, such as caulerpin, is highly potential compound as an anticancer (Permatasari et al., 2022b). Also, the safety profile of *C. racemosa* has been investigated using the MTT assay in another study, and in that study, the LC_50_ value for a macerated extract of *C. racemosa* (EM) in 24 h of incubation is 914.78 μg/ml. In terms of cytotoxicity, it was observed that *C. racemosa* was safe to be potentially developed into various products (Nurkolis et al., 2022).

### Cell migration with scratch wound healing assay

Cells were grown on 12-well plates and waited until they formed a monolayer, and they were scraped manually with a p10 micropipette tip and washed with media. The scratched cells were grown in starvation medium (FBS 0.5%) with various doses of *C. racemosa* extract (0, 50, 100, and 200 g/ml). Determination of the dose is based on the preliminary studies done previously. The wound closure process was observed at 0 and 24 h after scrapping cells. The scratched width was calculated using the ImageJ application (Permatasari et al., 2021).

### Observation of snail, vimentin, and E-Cadherin expression by immunofluorescence assay

Cell cultures were placed in 12-well-plates with the bottom of the wells fitted with a circular glass cover with a diameter of 18 mm (Matsunami, Japan). Cells were washed with PBS twice and then fixed in 4% formaldehyde in PBS for 15 min at room temperature. Afterwards, the cells were washed again with PBS twice and were permeabilized using incubation with 2 ml 0.1–0.5% Triton X-100 for 10 min in PBS at 4°C. Then, triton X-100 was aspirated and cells were washed three times with PBS. Cells were blocked with blocking buffer (10% goat serum, 2% bovine serum albumin, 0.2% Triton-X) for 1 h. Moreover, Snail observations was applied using anti-mouse Snail primary antibody, followed by anti-rabbit secondary antibody. Vimentin and E-Cadherin staining used anti-rabbit vimentin primary antibody 1:100 and anti-mouse E-cadherin primary antibody 1:100 followed by secondary antibodies, namely anti-mouse FITC (abcam) and anti-rabbit rhodamine (abcam) labeled with fluorochromes. Antibodies were diluted in blocking buffer and then incubated overnight at 4°C in a dark room. The samples then were washed 5 times with PBS, and the nuclei of the samples were incubated with 4,6-diamino-2-phenylindol (DAPI; Sigma Aldrich, Missouri, United States) 1 g/ml for 15 min and then washed with PBS 6 times. The sample was given mounting medium and then observed using an Olympus IX71 inverted fluorescence microscope with a magnification of ×200. Image quantification was performed using the ImageJ application (Sorrells et al., 2013; Permatasari et al., 2021).

### Data collection and statistical analysis

Data analysis used the application of Graphad Prism nine and SPSS version 22. The data were tested for normality and homogeneity using the Saphiro-Wilk test and Levene homogeneity test. If the data were normally distributed (*p* > 0.05), One-way ANOVA test was performed to test whether or not there were differences between the various treatment groups. If the results are significant (*p* < 0.05), the data will be continued with the Post Hoc test to see the significance between groups. If the data were not normally distributed (*p* < 0.05), the Kruskal-Wallis test will be performed to test whether there are differences between the various treatment groups. If the test results are significant (*p* < 0.05), the data will be continued with Dunn’s Post Hoc test to see the significance between groups. A correlation test was also carried out in this study. The results of data analysis are presented in the form of a diagram.

## Results and discussion

### Cell migration with scratch wound healing assay

**FIGURE 1:**
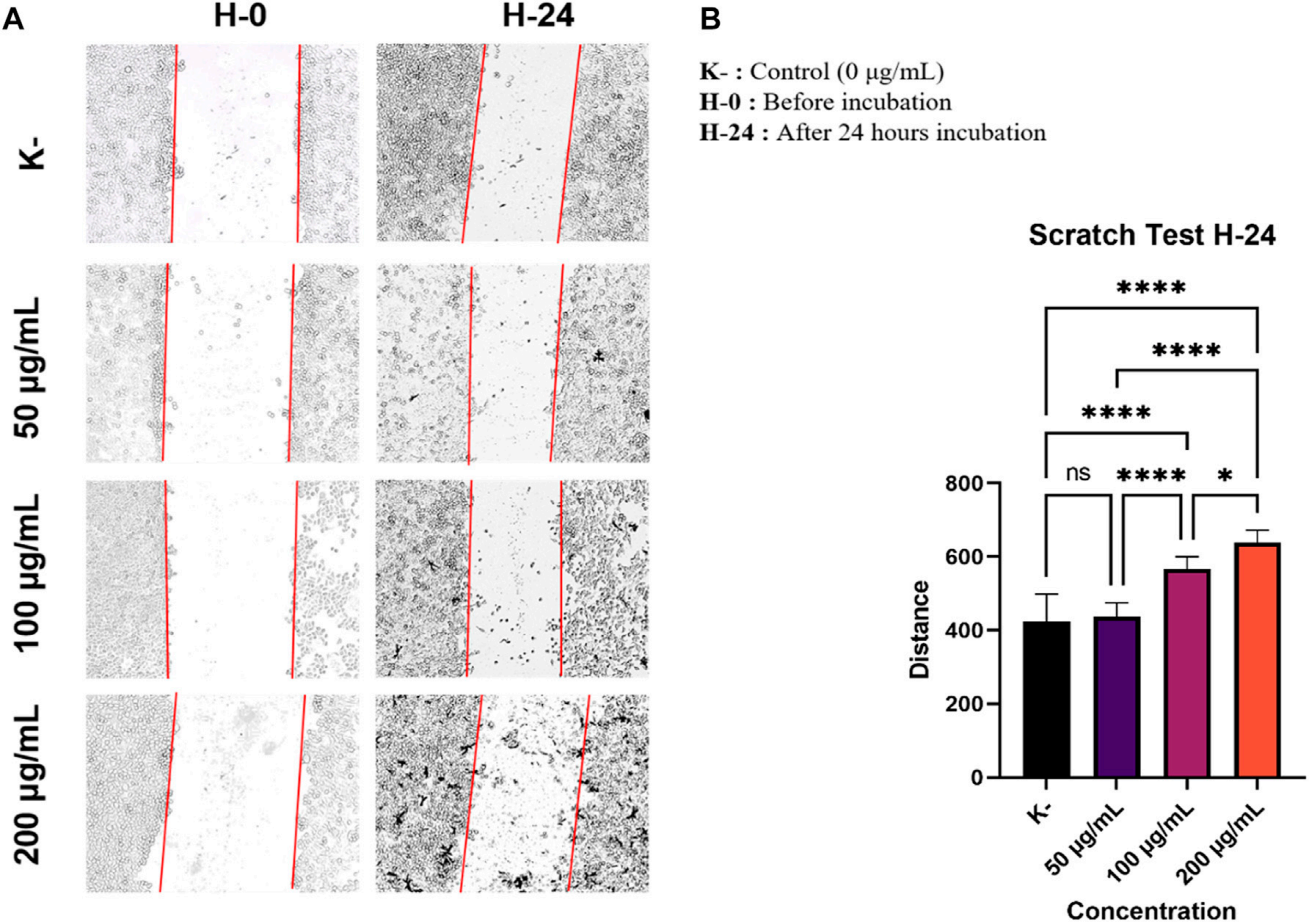
As the dose of *C. racemosa* increases, the distance between cells gets wider. **(A)** Morphology of HeLa cells after being treated with *C. racemosa* extract at various doses for 24 h **(B)** The stroke width of various doses of *C. racemosa* extract calculated using the ImageJ ImmunoRatio application, the data are presented in the form of mean ± standard deviation, *p < 0.05; ****p < 0.0001; ns: not significant (One-way ANOVA and Tukey’s multiple comparison test).

To investigate the anti-metastatic effect of *C. racemosa* extract on HeLa cancer cells, we did observation whether the *C. racemosa* extract affected cell migration or not. HeLa cells were grown to form a monolayer, then scraped and incubated with various concentrations of *C. racemosa* extract (0, 50, 100, and 200 g/ml) in starvation medium (low FBS 0.5%). Observation of scratch distance was done at 0 and 24 h after treatment using an inverted microscope was applied. The morphology of HeLa cells after being treated and incubated for 24 h is shown in Figure 1A. Distance measurements were carried out after 24 h of incubation, the distance was 423.9 ± 74.2 mm in the control group and the group treated with various doses of *C. racemosa* extract had a wider distance according to the order of the smallest dose, namely 437.3 ± 36.7 mm, 566.2 ± 33.5 mm, and 638.2 ± 33.9 mm. The results of this study obtained a significance value of 0.000 (sig <0.05) which indicates there is a significant difference between the treatment groups 24 h after treatment. In the Tukey test, there was a significant difference in the ratio of 0–100 g/ml and between 0 and 200 g/ml.

The ratio of the width of the scratches obtained from scratch wound healing assay test from each treatment group was calculated using the ImageJ ImmunoRatio application (Figure 1B). Overall, we found that the administration of *C. racemosa* extract inhibited HeLa cell migration in the group. There was a significant difference between the distance in the untreated group (0 g/ml) and the treatment group with doses of 100 g/ml and 200 g/ml, as it has been evidenced by the Pearson correlation test with r = 0.907 and a significance value of 0.000 (sig <0.05), this shows a significant positive correlation between the dose and the width of the scratch after incubation with *C. racemosa* extract for 24 h, which means that the higher the dose used will have a significant effect on the wider the stroke distance formed.

In this study, we investigated whether *C. racemosa* extract is potential of being anti-metastatic in HeLa cervical cancer cells. *C. racemosa* has been reported to contain various secondary metabolites such as caulerpin, caulerpenne (Cyn), caulersin, racemosin C, prismiterin, and pheophorbide-a as anticancer agents in various cancer cells (Chan et al., 2006; Lee et al., 2013; Ferramosca et al., 2016; Ridhowati and Asnani, 2016; Yu et al., 2017; Tanna et al., 2020). Research by Tanna et al (2020) revealed that Caulerpa spp could potentially to increase p53 gene expression in HeLa cells. The expression of BAX, which is also a regulator of apoptosis increased in all cell lines. In contrast, the expression of CDC2, which plays a role in tumor formation, is down-regulated about two-fold by *Caulerpa racemosa* extract (Tanna et al., 2020). This study is in line with other studies investigating the effect of the Caulerpin pigment isolated from the green algae of the Caulerpa genus on the migration of MDA-MB-231 breast tumor cells. Cell migration activity was observed by wound healing assay method. The study’s results showed that an inhibitory effect of cell migration was observed in cells treated with Caulerpin in both normoxia and hypoxia conditions (Liu et al., 2009). Unfortunately, until now the mechanism of *C. racemosa* extract as an anticancer in inhibiting migration and influencing the EMT process in HeLa cancer cells has not been found. This study demonstrated the anti-metastatic activity of *C. racemosa* extract from Indonesian waters in inhibiting cell migration. Migration inhibition was obtained by increasing the dose of *C. racemosa* extract, as indicated by the correlation value of the Pearson correlation test (r = 0.907). HeLa cells with a dose of 200 g/ml showed the most effective migration inhibition. This can be seen from the difference in the width of the scratches before and after treatment between the control group and the group treated with *C. racemosa* extract, with greater wound closure in the control group.

### Potency of *C. racemosa* extract as anti-metastatic to snail, vimentin, and E-Cadherin expression

**FIGURE 2:**
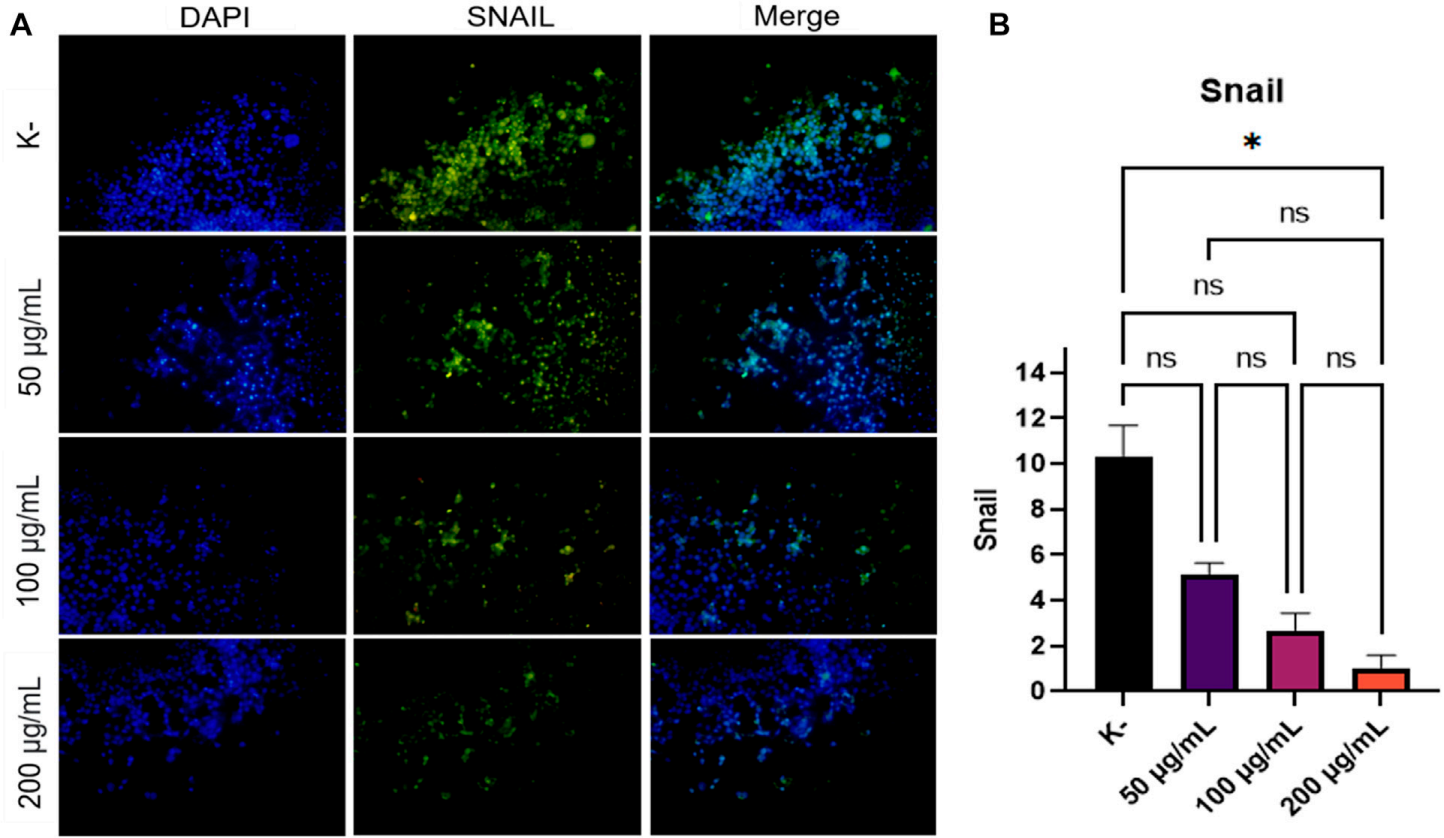
Decreased Snail expression after treatment with various doses of *C. racemosa* extract observed by immunofluorescence. **(A)** Expression of Snail (green) compared with DAPI (blue) on immunofluorescence assay. **(B)** Snail expression data are presented in terms of mean ± standard deviation, *p < 0.05; ns: not significant (Kruskall Wallis and Dunn’s multiple comparisons test).

EMT is a process involved in the process of cancer cell metastasis. Snail is one of the important factors that induce the occurrence of EMT, which is the initial process that occurs when cancer will metastasize. Determine the anti-metastatic activity of *C. racemosa*, Snail expression was observed in HeLa cell cultures treated with *C. racemosa* extract at several doses (0, 50, 100, and 200 g/ml). Nuclear staining using DAPI will show blue in the immunofluorescence image while cells expressing Snail will be green, as shown in Figure 2A. The quantification of luminescence of Snail expression was analyzed using the ImageJ application and shown in Figure 2B.

The findings showed that as the dosage of *C. racemosa* extract was increased, Snail expression in HeLa cell culture decreased. In the Kruskal-Wallis test, a *p*-value of 0.015 was obtained, which means that there was a significant difference in Snail expression between the four doses given. Significant differences were found mainly between the control dose and 200 g/ml, with a sig value of 0.013. Furthermore, the correlation test findings between *C. racemosa* dosage and Snail expression revealed a negative association with a very strong degree of correlation, precisely −0.973. This indicates that the higher the dose of *C. racemosa* given, the lower the amount of SNAIL expression.

The regulation of Snail expression is influenced by many factors in the tumor microenvironment. Notch’s intracellular domains, LOXL2, NF-B, HIF-1α, IKKα, SMAD, HMGA2, Egr-1, PARP-1, STAT3, MTA3, and Gli1 all interact directly with the Snail promoter to regulate Snail at the transcriptional level. Suppression of HIF-1α results in repression of Snail1 and EMT (Kaufhold and Bonavida, 2014). One of the bioactive compounds in *C. racemosa*, namely Caulerpin, is known to have the ability to inhibit HIF-1 activation (Liu et al., 2009).

**FIGURE 3:**
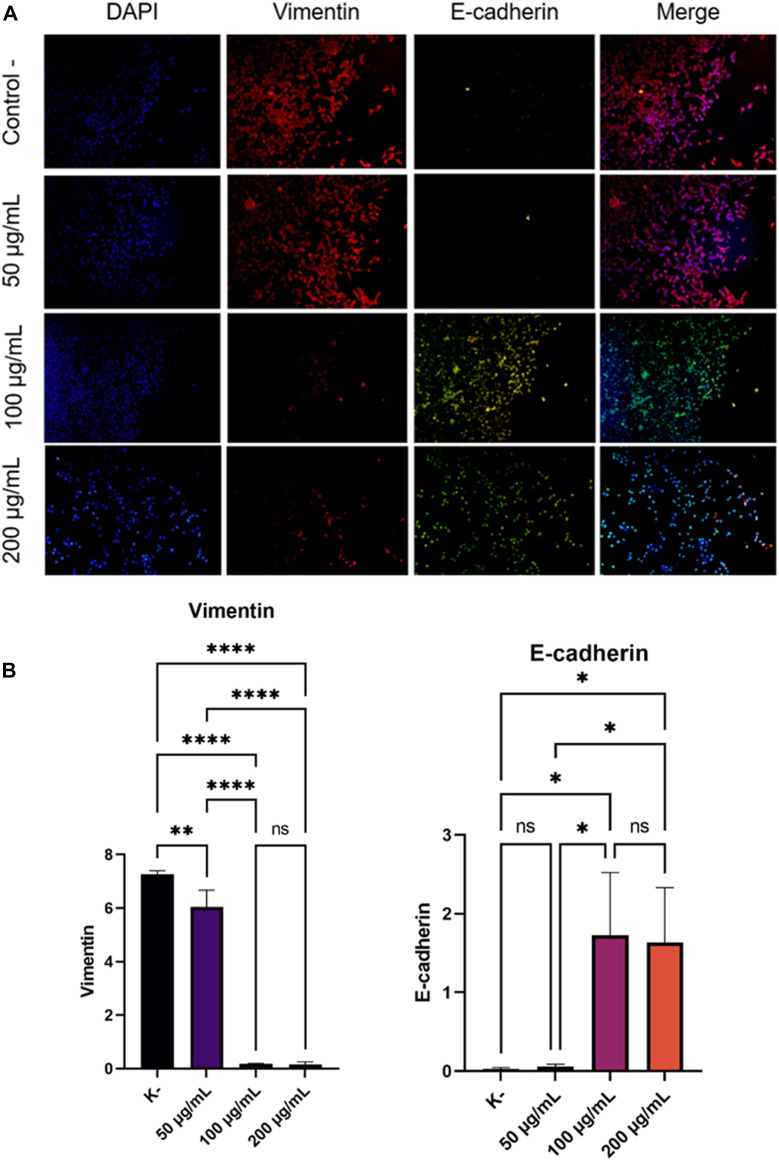
Decreased expression of Vimentin and increased expression of E-Cadherin after treatment with various doses of *C. racemosa* extract were observed with immunofluorescence. **(A)** Expression of Vimentin (red) and E-Cadherin (green) compared with DAPI (blue) on immunofluorescence assay. **(B)** Vimentin and E-Cadherin expression data are presented in terms of mean ± standard deviation, *p < 0.05; **p < 0.01; ****p < 0.0001; ns: not significant (One-way ANOVA and Tukey’s multiple comparison test).

Cancer cell metastases cannot be separated from the EMT process. Increased Vimentin expression and decreased E-Cadherin expression were associated with the EMT process. Therefore, we investigated the expression of Vimentin and E-Cadherin in HeLa cells that had been incubated with various doses (0, 50, 100, and 200 g/ml) of *C. racemosa* extract for 24 h. We evaluated the expression of Vimentin and E-Cadherin using the immunofluorescence method (Figure 3A). In the results of this study, we found that there was a significant decrease in the expression of Vimentin which is a mesenchymal marker (dose of 50, 100, and 200 g/ml) and a significant increase in the expression of E-Cadherin which is an epithelial marker (dose of 100, and 200 g/ml) as shown in Figure 3B. The decrease in the expression of Vimentin and the increase in the expression of E-Cadherin as evidenced by the Pearson correlation test with the results of the Vimentin test which showed that there was a significant negative correlation between the dose and the expression of Vimentin so that the higher the dose used, the lower the Vimentin expression (r = −0.869). Likewise, the Pearson correlation test on E-Cadherin expression shows there is a significant positive correlation between dose and E-Chaderin expression so that the higher the dose used will have a significant effect on the higher E-Cadherin expression (r = 0.826).

The occurrence of cancer cell metastatic cannot be separated from the EMT process (Qureshi et al., 2015). EMT is a process that causes loss of adhesion and polarity of epithelial initially attached to the basement membrane to cells that have a mesenchymal phenotype (Micalizzi et al., 2010). Changes in the epithelial cells cause the cells to become more motile and invasive. This process causes a decrease in epithelial markers, such as E-Cadherin, and an increase in mesenchymal markers, such as Vimentin (Qureshi et al., 2015). Loss of E-Cadherin expression is associated with increased EMT processes and often occurs in tumor metastases so that EMT processes usually do not occur when E-Cadherin expression is increased in cancer cells (Permatasari et al., 2021). Our results showed an increase in E-Cadherin expression in HeLa cells treated with *C. racemosa* extract (Figure 3B). This indicates the inhibition of the EMT process by the administration of the *C. racemosa* extract.

After the EMT process is finished, cells then acquire a mesenchymal phenotype and express mesenchymal markers such as Vimentin (Permatasari et al., 2021). Our study showed the decrease Vimentin expression in HeLa cells treated with *C. racemosa* extract. Vimentin expression decreased significantly at concentrations of 100 and 200 g/ml (Figure 3B). These results indicate that *C. racemosa* extract inhibited the EMT process in HeLa cervical cancer cells.

Snail is one of the EMT regulators that can suppress the expression of E-Cadherin protein by binding to its promoter. Snail also controls the proteolytic activity of matrix metalloproteinases associated with EMT processes and stromal invasion, thereby causing upregulation of mesenchymal markers such as Vimentin and Fibronectin (Permatasari et al., 2021). From the results of our research, *C. racemosa* extract inhibited Snail expression, thereby increasing E-cadherin expression and decreasing Vimentin expression. Thus, *C. racemosa* extract is thought to be able to be an anti-metastatic agent in HeLa cancer cells.

### Strengths and limitations

The strength of this study is that it can demonstrate that *C. racemosa* extract can prevent HeLa cell migration and precisely explore how it does so by downregulating Snail and Vimentin expressions and upregulating E-Cadherin expression. The limitation of this study is that it still needs to carry out *in vitro* on HeLa cell cultures with a limited sample, raising the possibility that additional variables could impact on the results if they were tested on actual living organisms. Moreover, research has not been able to pinpoint the exact component of *C. racemosa* that has these effects or fully explain how it works to limit protein expression. Additional research is required to make *C. racemosa* one of the therapeutic agents of choice for treating cancer in the future, such as finding out more about the optimal dose of *C. racemosa* to inhibit cancer metastasis in living organisms how to turn it into an accessible product.

## Conclusion and practical implications

The results of this study indicate the anticancer activity of *Caulerpa racemosa* through anti-metastatic by significantly inhibiting the migration of HeLa cancer cells, reducing Snail as an inducer of the EMT process and the expression of the mesenchymal marker Vimentin, and significantly increasing the epithelial marker E-Cadherin. These results indicate that *C. racemosa* can potentially to be an anticancer candidate by inhibiting of cell migration and the EMT process so that metastatic from cancer cells can be inhibited. Further studies are needed to determine the mechanism of *C. racemosa* extract from Indonesian waters as an anti-metastatic HeLa cervical cancer cell.

## Data Availability

The original contributions presented in the study are included in the article/Supplementary Material, further inquiries can be directed to the corresponding author.

## Abbreviations

BAX, Bcl-2-associated X protein; CDC2, Cell-Division Cycle 2; Cyn, Caulerpenyne; DAPI, 4,6-diamino-2-fenilindol; DMEM, Dulbecco’s Modified Eagle Medium; DMSO, Dimethyl sulfoxide; EDTA, Asam Etilendiamintetraasetat; EGF, Endothelial Growth Factor; Egr-1, Early Growth Response 1; EMT, Epithelial-Mesenchymal Transition; FBS, Fetal Bovine Serum; FITC, Fluorescein isothiocyanate; Gli1, Glioma-associated Oncogene Homologue 1; HIF-1α, Hypoxia-inducible Factor 1-alpha; HMGA2, High Mobility Group AT-Hook 2; HPV, Human Papilloma Virus; IKKα, IκB Kinase α; LOXL2, Lysyl Oxidase Homolog 2; MTA3, Metastatic Tumor Antigen 3; NF-B, Nuclear Factor Kappa B; PARP-1, Poly [ADP-ribose] Polymerase 1; PBS, Phosphate Buffer Saline; STAT3, Signal Transducer and Activator of Transcription 3.
